# Multi-index analysis of climate change events recorded by loess-paleosol deposits in the upper Hanjiang River valley since MIS 3

**DOI:** 10.1371/journal.pone.0341061

**Published:** 2026-03-04

**Authors:** Wentong Zhang, Lansong Lv, Jiayu Lu, Ye Lv, Haiyan Wang, Xu Xu, Jiangli Pang

**Affiliations:** 1 Institute of Jiangsu Coastal development, Yancheng Teachers University, Yancheng, Jiangsu, China; 2 School of Geography and Tourism, Shaanxi Normal University, Xi’an, Shaanxi, China; 3 Shandong Provincial Territorial Spatial Ecological Restoration Center, Jinan, Shandong, China; 4 School of Resources and Environment, Linyi University, Linyi, Shandong, China; 5 Development Research Center for Natural Resource and Real Estate Assessment, Shenzhen (Center for Environmental Monitoring of Geology), Shenzhen, Guangdong, China; 6 Shenzhen Institutes of Advanced Technology, Chinese Academy of Sciences, Shenzhen, Guangdong, China; 7 Colleges of Education, Linyi University, Linyi, Shandong, China; Institute of Earth and Environment, Chinese Academy of Sciences, CHINA

## Abstract

The upper Hanjiang River basin has been an important area for human life and production since ancient times. Marine Isotope Stage 3 (MIS 3) is a special period of relatively warm and humid climate during the last glacial period. However, the climate record of MIS 3 in this region, especially the difference of chemical weathering characteristics between this region and the northern Loess Plateau, remains unclear. An in-depth field investigation was conducted in this study on the upper Hanjiang River valley and we found a typical loess-paleosol profile named Tuojiawan (TJW). Multi-proxy indicators including sedimentology, chronology, magnetic susceptibility, grain size, and geochemistry were used to analyze the climate change characteristics. The results show that the stratigraphic consists of fluvial deposits (T_1_-al1), interaction layer (T_1_-al2), Malan loess (L_1-3_), paleosol (L_1-S2_), Malan loess (L_1-2_), paleosol (L_1-S1_), Malan loess (L_1-1_), transitional loess (L_t_), paleosol (S_0_), recent loess (L_0_), and modern soil (MS). The pedogenic intensity varies significantly in different layers and presents a tendency of S_0_ > L_1-S2_ > L_1-S1_ > L_t_ > L_1_ (L_1-1_, L_1-2_, L_1-3_). This indicates that MIS 3 is not a continuously dry and cold stage. TJW profile also showed a phase of gradual shift to warm-wet (11.5–8.5 ka BP), maximum warm-wet period (8.5–3.1 ka BP), and a phase of gradual shift to cool-dry (after 3.1 ka BP). Compared with the records of the Loess Plateau, the chemical weathering intensity of the warm and humid event in the upper reaches of the Hanjiang River in the late MIS 3 is different, which reveals the unique response mode of the region to global climate change and may be controlled by different monsoon subsystems.

## 1. Introduction

Loess-paleosol sequence is considered to be one of the best terrestrial archives for recording paleo-environmental evolution [1]. Many Loess Plateau profiles recorded a relatively warm-wet climate stage in the last glacial period [1–6], which was significantly in contrast to the dry-cold climate, and the temperature and humidity degree was lower than that of the Holocene and the last interglacial period [3,7], occurrence time of which may correspond to MIS 3 period [7,8]. This relatively warm-wet climate change event has also been recorded in loess profiles in the middle and lower reaches of the Yangtze River [9]. Although climate during MIS 3 was relatively warm and wet, as recorded in many loess profiles, the changing characteristics recorded by various profiles were still quite different [3,7,8], as different classification standards for climate change may lead to different divisions of cool and warm stages, while the late and early temperature and humidity in MIS 3 are consistent. Further theoretical verification of these issues is needed.

Located at the southern foot of the Qinling Mountains in China, the Hanjiang River lies within the transition zone between the temperate and subtropical monsoon climates, making it highly sensitive to climate change. In recent years, the spatial distribution, stratigraphy, chronology and characteristics of pedogenesis of the Hanjiang River valley loess have been studied deeply [5,6,10–12]. However, the climate change record during MIS 3 in this area has not been studied yet. It is also important to examine whether these climate changes are regional or global. In this study, we focused on Tuojiawan (TJW) loess-paleosol profile in the upper Hanjiang River valley. Through a comprehensive study of its magnetic susceptibility, grain size, geochemical elements, with optical stimulated luminescence (OSL) age, and aimed at: (1) using alternative climate indicators to study the process of climate change in the upper reaches of the Hanjiang River since MIS 3; (2) comparing with climate records from different regions, and obtaining the regional climate change records since the end of the Late Pleistocene period.

## 2. Geographical setting

**Fig 1:**
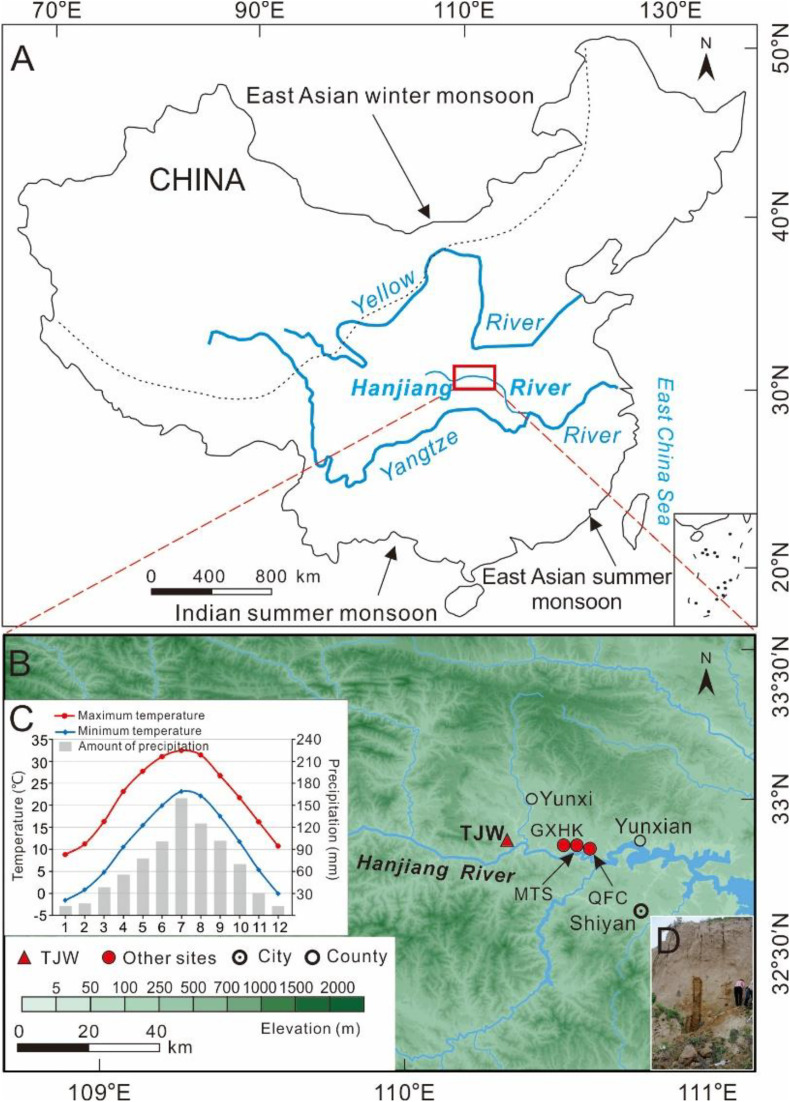
The study area. **(A)** The location of the upper Hanjiang River drainage basin between the Yunxi and Yunxian Reaches (the area enclosed in red lines). **(B)** The location of the TJW profile and other comparison profile sites (after https://www.gscloud.cn) [5,6] within the Yunxian Basin in the reaches of the Hanjiang River valley. **(C)** Monthly average temperature and precipitation in Yunxi County from 1981 to 2010 (after https://www.nmc.cn). **(D)** Photo of TJW profile.

The Hanjiang River, a major tributary of the Yangtze River in China, flows through Shaanxi and Hubei provinces in central China (Fig 1A). The Hanjiang River has a length of 1577 km and a catchment area of 1.59 × 10^5^ km^2^. The mean annual runoff is 5.17 × 10^10^ m^3^ and the mean suspended sediment load is 2.5 kg/m^3^. The upper reach of the mainstream (Danjiangkou Reservoir upstream) is 925 km long with a drainage area of 9.53 × 10^4^ km^2^ and an average annual runoff of 3.79 × 10^10^ m^3^ [13]. In addition, the upper Hanjiang River plays an important role in furnishing the main water source for the Middle Route Project of the South-North Water Transfer Project of China [14].

The upper Hanjiang River flows from west to east between the Qinling and Dabashan Mountains and cuts into granitic and metamorphic rocks, forming deep and narrow bedrock gorges with some large and small basins along the mainstream of the Hangjiang River [15]. Three visible alluvial terraces, which are 10–15 m, 30–40 m, 60–70 m above present river water level respectively, have been identified in the basins of the upper Hanjiang River [10,16]. In exposed riverbank cliffs, eolian loess-paleosol sequences were often found overlying past fluvial deposits such as floodplain sediments and gravels [5,13].

The upper Hanjiang River is located in the humid region with a subtropical monsoonal environment that is extremely sensitive to climate change. Mean annual temperature ranges from 14–16 °C and mean annual precipitation from 800–900 mm, which is affected by the warm and humid air currents of East Asian summer monsoon and Indian summer monsoon (Fig 1A). Over 75% of precipitation occurs during May and October due to the special hydro-climate conditions of high seasonal variability.

## 3. Materials and methods

### 3.1. Materials

**Fig 2:**
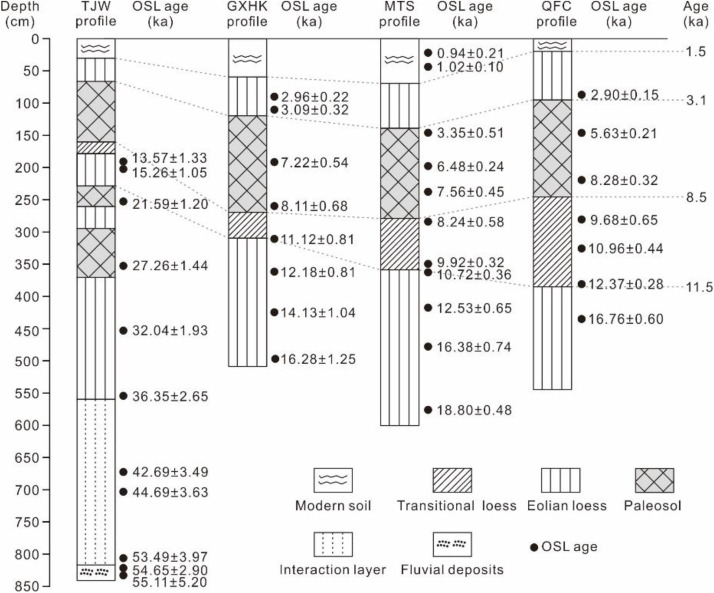
Pedostratigraphy and chronology of the TJW profile [17,22] and other comparison profiles (GXHK, MTS, QFC) [5,6] from the first terrace of the Yunxian Basin in the upper reaches of the Hanjiang River valley.

Extensive field investigations were carried out on the first terrace in the Yunxian Basin from 2010 to 2014. Detailed geomorphological, pedological, sedimentological and stratigraphic observations were performed throughout the Yunxian Basin. A complete loess-paleosol sequence that had not been disturbed by human activities or soil erosion processes was discovered at the village of TJW in the town of Guanyin, Yunxian county, Hubei Province, China (TJW profile, 32°05’47“N, 110°22’51”E, 168 m asl, Fig 1B). The pedostratigraphy of the TJW profile from bottom to top is listed as fluvial deposits (T_1_-al1) →Interaction layer (T_1_-al2) → Malan Loess (L_1-3_) →Paleosol (L_1-S2_) →Malan Loess (L_1-2_) →Paleosol (L_1-S1_) →Malan Loess (L_1-1_) →Transitional loess (L_t_) →Paleosol (S_0_) →Recent loess (L_0_) →Modern soil (MS). The detailed stratigraphic description at the TJW profile is shown in Table 1. The stratigraphic characteristics of TJW profile are basically the same as those of other loess profiles in the Hanjiang River basin, such as Guixianhekou (GXHK) profile, Mituosi (MTS) profile and Qianfangcun (QFC) profile in Yunxian County [5-6,12], as shown in Fig 2. The TJW profile provides a well-preserved record of regional climate evolution since 55 ka BP.

**Table 1:** Stratigraphic description of the Tuojiawan (TJW) profile in the upper Hangjiang River valley.

Depth (cm)	Pedostratigraphic subdivisions	Pedosedimentary description
0-30	Modern soil (MS), Unit 1	Pale brown (7.5YR5/3), clay-silt texture, friable and porous, some plant roots
30-66	Recent loess (L_0_), Unit 2	Pale yellow orange (10YR7/4), silt texture, blocky structure
66-160	Paleosol (S_0_), Unit 3	Bright brown-reddish brown (5YR5/6–5YR5/4), clay texture, ribbed block structure
160-178	Transitional loess (L_t_), Unit 4	Cloudy yellow orange (10YR6/4), silt texture, massive structure
178-228	Malan Loess (L_1-1_), Unit 5	Pale yellow orange (10YR7/4), silt texture, uniform massive structure
228-260	Paleosol (L_1-S1_), Unit 6	Dark reddish brown (5YR3/6), clayey silt texture, ridged massive structure
260-294	Malan Loess (L_1-2_), Unit 7	Pale yellow orange (10YR7/4), silt texture, uniform massive structure
294-370	Paleosol (L_1-S2_), Unit 8	Dark reddish brown (5YR3/6), clayey silt texture, ridged massive structure
370-560	Malan Loess (L_1-3_), Unit 9	Pale yellow orange (10YR7/4), silt texture, uniform massive structure
560-818	Interaction layer (T_1_-al2), Unit 10	Loess-sand interlayer, loess being cloudy yellow orange (10YR7/4), silt-fine sand texture; sand layer being fine sand texture
818-838	Fluvial deposits (T_1_-al1), Unit 11	Pale yellow brown (10YR5/4), dual structure, fluvial sand in the upper part and gravel fluvial in the lower part

### 3.2. Methods

After pedo-stratigraphic subdivisions, 353 sediment samples were collected at 2 cm intervals from 0 to 570 cm and at 4 cm intervals from 570 to 838 cm in the TJW profile. All the experiments were carried out in the Environment Change Laboratory of Shaanxi Normal University. Magnetic susceptibility was measured using 10 g of sample with a Bartington MS2 magnetic susceptibility meter (0.47/4.7 kHz). The grain size distribution of the samples were analyzed using a Beckman Coulter laser diffraction particle size analyzer with dispersant (Calgon) after digestion with 10% H_2_O_2_ and 10% HCl to remove organic matter and carbonates, respectively [17]. Geochemical element analysis was performed using a PANalytical PW2404 X-ray fluorescence spectrometer.

Samples for optically stimulated luminescence (OSL) dating were taken with a steel cylinder from the profile and immediately packed with aluminum foil during the fieldwork. The OSL measurements were performed in the OSL laboratory at Shaanxi Normal University. OSL dating on quartz grains (90–125 μm) was performed using the single-aliquot regenerative (SAR) dose protocol [18]. The preheat and cut-heat temperatures were 260 °C and 220 °C, respectively. Here, 12 aliquots were measured for each sample. The equivalent dose of all aliquots was measured with an automated Risø–TL/OSL DA–20 DASH Reader with a combined blue (470 nm, 50 mW/cm^2^) and infrared (875 nm, 150 mW/cm^2^) light-emitting diode (LED) unit, and a ^90^Sr/^90^Y beta source for irradiation [19]. U, Th, and K concentrations were measured using neutron activation analysis (NAA) at the China Institute of Atomic Energy. Effective dose rate was calculated from elemental concentrations using revised dose rate conversion factors [20]. The computer program Age.exe was used to calculate OSL dates [21]. In order to ensure the accuracy and reliability of the OSL data of the TJW profile, the distribution of the single De value, the dispersion of the natural OSL signal of the sample, and the dispersion of the OSL signal of the first regeneration dose of the sample were analyzed [17,22]. The age-depth relationship was determined using the interpolation method.

## 4. Results

### 4.1. Lithology and chronology in TJW profile

**Fig 3:**
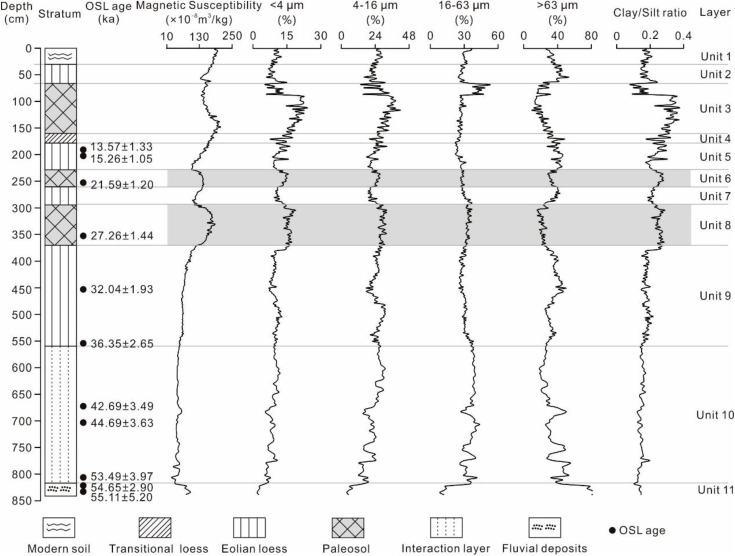
Pedostratigraphy, magnetic susceptibility and grain size distribution in the loess-paleosol sequences at the TJW profile in the upper Hanjiang River valley [17].

Eleven lithological units were identified from the base upwards in the TJW profile (Figs 2, 3), as described in Table 1. Reliable chronology is vitally important for reconstructing paleoclimatic and pedogenic environmental changes, as well as for correlating with other dated records on a time-scale [23]. The ages of loess-paleosol deposits in the upper Hanjiang River are obtained with OSL dating. OSL ages in loess-paleosol profiles at TJW, GXHK, MTS, and QFC sites are presented in Fig 2. The loess-paleosol sequence overlies T_1_-al1 in the basal part of the TJW profile. OSL samples from the top part of the T_1_-al1 were dated to 55.11 ± 5.20 ka and 54.65 ± 2.90 ka. The sample from the bottom part of the T_1_-al2 was dated to 53.48 ± 3.97 ka. The sample from the bottom part of L_1_ was dated to 53.95 ± 3.84 ka (Figs 2 and 5). Thus, the boundary between T_1_-al1 and T_1_-al2 can be constrained to 55.0 ka BP, marking the uplift period of the first terrace of the Hanjiang River and the beginning accumulation of the Malan loess. OSL samples at the bottom of the L_1-S2_ layer were dated to 27.26 ± 1.44 ka, and at the bottom of L_1-S1_ layer was dated to 21.59 ± 1.20 ka. Therefore, the results indicates that these two layers were formed about 27.26 ± 1.44 ka BP and 21.59 ± 1.20 ka BP, respectively. OSL samples from the top part of Malan loess (L_1-1_) were dated to 15.26 ± 1.05 ka (200–205 cm) and 13.57 ± 1.33 ka (190–195 cm). Similar results were observed in the GXHK profile (Fig 2). The L_1_ is directly covered by the transitional loess (L_t_) of the early Holocene. Previous studies have reported that the bottom of the L_t_ was dated to 10.72 ± 0.39 ka in the MTS profile, and 12.37 ± 0.28 ka in the QFC profile [5,6]. Therefore, the boundary between L_1_ and L_t_ can be confined to 11.5 ka BP in the TJW profile.

Paleosol (S_0_) is widely underlain by L_t_ of the early Holocene and is buried by recent loess (L_0_) and Modern soil (MS) of the late Holocene. OSL samples from the top of the L_t_ were dated to 8.24 ± 0.58 ka in the MTS profile, and 9.68 ± 0.65 ka in the QFC profile. OSL samples from the bottom part of S_0_ were dated 8.11 ± 0.68 ka in the GXHK profile, and 8.28 ± 0.32 ka in the QFC profile. The boundary between L_t_ and S_0_ can be confined to 8.5 ka BP. OSL samples from the top of S_0_ were dated to 3.35 ± 0.51 ka in the MTS profile, and from the bottom of L_0_ were dated to 3.09 ± 0.32 ka in the GXHK profile, and 2.90 ± 0.15 ka in the QFC profile. Thus, the major boundary between S_0_ and L_0_ can be confined to 3.1 ka BP.

### 4.2. Vertical distribution of magnetic susceptibility, grain size, loss-on-ignition and geochemical elements in TJW profile

**Fig 4:**
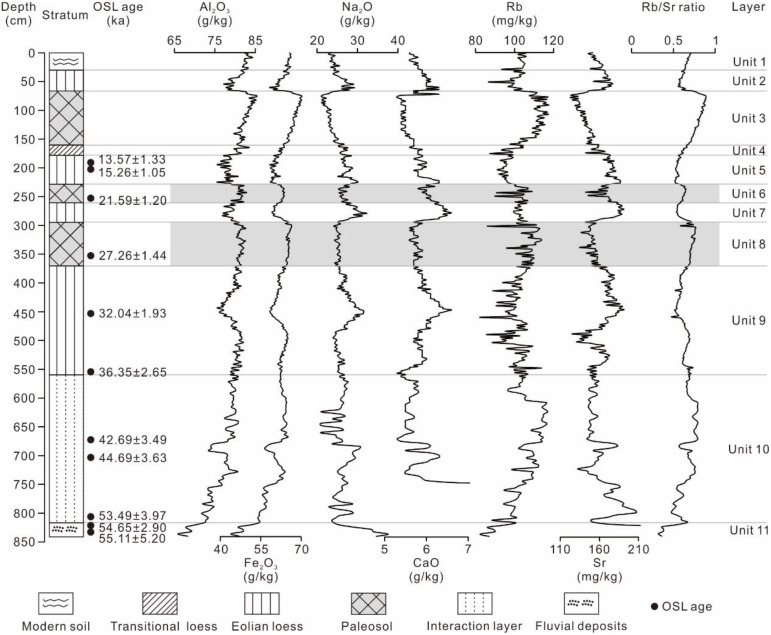
Pedostratigraphy, geochemical elements features in the loess-paleosol sequences at the TJW profile in the upper Hanjiang River valley.

Magnetic susceptibility, grain size composition, and geochemical element changes are consistent with lithological changes (Figs 3, 4) and are described as follows:

Unit 11 (838–818 cm; > 55.0 ka BP). The magnetic susceptibility decreases upward, the sand (>63 μm) exhibits the same trend, i.e., 77.60 × 10^−8^–97.40 × 10^−8^ m^3^·kg^-1^ and 74.87–85.81%, while clay (<4 μm) and silt (4–63 μm) exhibit the opposite trend (Table 2, Fig 3). Al_2_O_3_, Fe_2_O_3_, Rb, and Rb/Sr contents increase upward, while Na_2_O and Sr contents exhibit opposite trends (Table 3, Fig 4).

**Table 2:** Average values of magnetic susceptibility and grain-size distribution in Tuojiawan (TJW) profile in the upper Hanjiang River valley [17].

Stratigraphy	Magnetic susceptibility (×10^-8^m^3^·kg-1)	< 4 μm (%)	4-16μm (%)	16-63μm (%)	>63μm (%)	Clay/silt ratio
MS (0–30 cm)	186.62	10.16	26.21	31.35	32.29	0.18
L_0_ (30–66 cm)	156.88	8.80	22.38	28.20	40.62	0.17
S_0_ (66–160 cm)	166.15	17.24	31.09	30.67	21.00	0.28
L_t_ (160–178)	173.03	13.18	25.83	24.87	36.12	0.26
L_1-1_ (178–228)	130.67	11.12	22.74	25.55	40.58	0.23
L_1-S1_ (228–260)	139.08	13.81	25.15	28.23	32.81	0.26
L_1-2_ (260–294)	121.49	11.30	21.98	31.33	35.38	0.21
L_1-S2_ (294–370)	162.59	15.86	29.00	33.14	21.99	0.26
L_1-3_ (370–560)	76.15	9.99	17.60	29.94	34.59	0.18
T_1_-al2 (560–818)	51.09	8.71	22.68	36.14	32.47	0.15
T_1_-al1 (818–838)	86.00	2.47	5.63	11.96	79.94	0.14

**Table 3:** Average values of geochemical elements in Tuojiawan (TJW) profile in the upper Hanjiang River valley.

Stratigraphy	Al_2_O_3_ (g/kg)	Fe_2_O_3_ (g/kg)	Na_2_O (g/kg)	CaO (g/kg)	Rb (mg/kg)	Sr (mg/kg)	Rb/Sr
MS (0–30 cm)	82.6	65.3	24.0	5.7	103.3	156.0	0.66
L_0_ (30–66)	79.8	62.3	26.4	6.0	100.1	167.1	0.60
S_0_ (66–160)	82.5	67.2	23.2	5.5	111.1	140.1	0.80
L_t_ (160–178)	80.5	62.3	26.7	5.8	97.3	159.1	0.61
L_1-1_ (178–228)	78.0	60.0	27.5	5.9	95.5	175.8	0.54
L_1-S1_ (228–260)	81.2	63.0	26.5	6.0	102.5	166.0	0.62
L_1-2_ (260–294)	78.8	61.2	28.9	6.3	102.2	177.6	0.58
L_1-S2_ (294–370)	81.2	65.2	25.2	5.7	107.3	150.3	0.71
L_1-3_ (370–560)	79.7	62.1	26.7	5.9	100.6	164.9	0.61
T_1_-al2 (560–818)	77.1	60.6	26.1	10.5	106.1	160.1	0.67
T_1_-al1 (818–838)	67.9	46.8	33.8	25.2	86.9	234.5	0.36

Unit 10 (818–560 cm; 55.0–36.0 ka BP). Magnetic susceptibility is the lowest value of the whole profile, i.e., 51.09 × 10^−8^ m^3^·kg^-1^ (Table 2), and it increases gradually from bottom to top (Fig 3). The clay content and clay/silt ratio exhibit low values. Fine silt (4–16 μm) and coarse silt (16–63 μm) contents increase markedly compared with unit 11 (Fig 3). The contents of Al_2_O_3_, Fe_2_O_3_, Rb, and Rb/Sr ratio exhibit the same trend with fine silt, increase markedly compared with unit 11, while Na_2_O and Sr show opposite trends (Fig 4).

Unit 9 (560–370 cm). The magnetic susceptibility and clay/silt ratio increase slightly compared to unit 10, i.e., 76.15 × 10^−8^ m^3^·kg^-1^ and 0.18 (Table 2). The contents of clay, silt and geochemical element are basically the same with unit 10 (Tables 2 and 3; Figs 3 and 4).

Unit 8 (370–294 cm). Magnetic susceptibility (162.59 × 10^−8^ m^3^·kg^-1^), clay content (15.86%), fine silt (29%), and clay/silt ratio (0.26) increase significantly compared to unit 9. Sand content shows an opposite trend of 21.99% (Table 2, Fig 3). Al_2_O_3_, Fe_2_O_3_, Rb, and Rb/Sr contents exhibit the same trend with clay and fine silt, with unit 8, while Na_2_O, CaO and Sr contents exhibit opposite trends (Table 3, Fig 4).

Unit 7 (294–260 cm). Magnetic susceptibility, the contents of clay, fine silt, and clay/silt ratio decrease markedly compared to unit 8. In contrast, the sand content exhibits the opposite trend (Table 2, Fig 3). The contents of Al_2_O_3_, Fe_2_O_3_, Rb, and Rb/Sr ratio exhibit the same trend as clay and fine silt, increasing with unit 7, while the contents of Na_2_O, CaO and Sr exhibit opposite trends (Table 3, Fig 4).

Unit 6 (260–228 cm). Magnetic susceptibility, the contents of clay, fine silt, and clay/silt ratio increase markedly compared to unit 7, slightly lower than unit 8 (Fig 3). In contrast, the sand content exhibits the opposite trend (Fig 3). The contents of Al_2_O_3_, Fe_2_O_3_, Rb, and Rb/Sr ratio exhibit the same trend with clay and fine silt, increasing with unit 7, while the contents of Na_2_O, CaO and Sr exhibit opposite trends (Table 3; Fig 4).

Unit 5 (228–178 cm; ~ 20–11.5 ka BP) is very similar to units 7 and 9 (Fig 3).

Unit 4 (178–160 cm; 11.5–8.5 ka BP). Magnetic susceptibility, contents of clay, fine silt, and clay/silt ratio increase compared to unit 5 (Fig 3). The contents of Al_2_O_3_, Fe_2_O_3_, Rb, and Rb/Sr ratio exhibit the same trend as clay and fine silt, increasing with unit 5, while the contents of Na_2_O, CaO and Sr exhibit opposite trends (Table 3, Fig 4).

Unit 3 (160–66 cm; 8.5–3.1 ka BP). Magnetic susceptibility, the contents of clay, fine silt, coarse silt, and clay/silt ratio increase markedly compared to unit 4, are the high value of the whole profile, i.e., 166.15 × 10^−8^ m^3^·kg^-1^, 14.24%, 31.09%, 30.67%, and 0.28, the sand content varies little within the unit, only 21% (Table 2, Fig 3). The contents of Al_2_O_3_, Fe_2_O_3_, Rb, and Rb/Sr ratio exhibit the same trend with clay and fine silt, increasing markedly with unit 4, while the contents of Na_2_O, CaO and Sr exhibit opposite trends (Table 3, Fig 4).

Unit 2 (66–30 cm; 3.1–1.5 ka BP). Magnetic susceptibility, the contents of clay, fine silt, coarse silt, and clay/silt ratio decrease compared to unit 3, sand content increases markedly with unit 3 (Table 2, Fig 3). The contents of Al_2_O_3_, Fe_2_O_3_, Rb, and Rb/Sr ratio exhibit the same trend with clay and fine silt, decreasing with unit 4, while the contents of Na_2_O, CaO and Sr exhibit opposite trends (Table 3, Fig 4).

## 5. Discussion

### 5.1. Pedogenic intensity change in the TJW profile

Magnetic susceptibility is closely associated with the enrichment of fine-grained ferromagnetic minerals in loess-paleosol profiles [24,25]. It has been widely used as a proxy to reflect pedogenic intensity and paleoclimatic and environmental changes in semi-arid and semi-humid climate regions [26–29]. Generally, higher MS values indicate intensive pedogenesis and a warm and wet climate, while lower values imply weaker pedogenesis and a cold and dry climate [1,30–32]. The magnetic susceptibility values of the TJW profile range from 29.40 × 10^-8^ to 207.80 × 10^-8^ m^3^·kg^-1^. The S_0_ layer shows a relatively high value (166.15 × 10^-8^ m^3^·kg^-1^), indicating intensive pedogenesis and the formation of abundant secondary ferromagnetic minerals during 8.5–3.1 ka BP. This suggests warm and humid climate conditions. This is consistent with the middle Holocene Megathermal period [33]. Magnetic susceptibility values in loess (L_1-1_, L_1-2_, L_1-3_) are relatively low, indicating weak pedogenesis and a cold and dry climate. It is noteworthy that in the Malan Loess, the magnetic susceptibility shows one peak around 27 ka and another one around 21 ka (Table 2, Fig 3), with mean values of 139.08 × 10^-8^ m^3^·kg^-1^ and 162.59 × 10^-8^ m^3^·kg^-1^, respectively. These values are higher than those of L_1_(L_1-1_, L_1-2_, L_1-3_), indicating that these soils underwent a certain degree of pedogenesis and represent weakly developed paleosols. However, the magnetic susceptibility values of L_1-S1_ and L_1-S2_ are lower than those of S_0_, indicating weaker soil formation compared with paleosol S_0_.

Grain size distribution characteristics of loess are widely used as a surrogate indicator of East Asian monsoon changes [1,4,34–36]. Generally, the content of sand (>63 μm) is used to reflect the rise and fall of the winter monsoon, and the content of clay (<4 μm) indicates the pedogenesis and strength of the summer monsoon [3,37]. The results show that the grain size composition of TJW profile is mainly silt (4–63 μm). In general, the peak clay content occurs in S_0_ (17.24%), whereas lower mean values (11.12%, 11.30%, 9.99%) are observed in the loess layers (L_1-1_, L_1-2_, L_1-3_). Sand content exhibits the opposite trend to clay in S_0_ and L_1_ layers. These characteristics indicate that the S_0_ underwent intense weathering and pedogenesis and unstable minerals were decomposed to form a large number of fine secondary clay minerals, while loess (L_1-1_, L_1-2_, L_1-3_) underwent weak pedogenesis. At the same time, the grain-size variation curve also shows two distinct peaks around 27 ka and 21 ka (Fig 3), corresponding to the weak paleosol layers L_1-S1_ and L_1-S2_. The content of clay (mean value: 13.81% and 15.86%) in the paleosol layer (L_1-S1_ and L_1-S2_) is significantly higher than that of loess (L_1-1_, L_1-2_, L_1-3_), but lower than that of S_0_ (mean value: 17.24%, Table 2), and sand content shows the opposite pattern. These characteristics indicate that the weak paleosols underwent noticeable pedogenic alteration. However, their pedogenic intensity is weaker than that of S_0_.

The abundances of geochemical elements in aeolian deposits provide critical information on their provenance and on evolutionary characteristics related to weathering and pedogenic intensity [1,38–40]. The enrichment of major elements Al_2_O_3_ and Fe_2_O_3_ is associated with the formation of secondary clay minerals during weathering and pedogenesis [41]. The enrichment of Rb in paleosol indicates a warm and humid climate, while the higher content of Sr in the loess layers suggests a dry and cold climate [42,43]. The curves of these elements (Al_2_O_3_, Fe_2_O_3_, Rb) present a similar pattern to the curve of clay content (Figs 3 and 4). Higher concentrations occur in S_0_ (mean value: 82.5 g/kg, 67.2 g/kg, 111.1 mg/kg) whereas lower concentrations occur in the loess layers (L_1-1_, L_1-2_, L_1-3_). Additionally, the geochemical elements (Al_2_O_3_, Fe_2_O_3_ and Rb) variation curves show two obvious peaks around 27 ka and 21 ka (Fig 4), corresponding to the weak paleosol layers (L_1-S1_ and L_1-S2_). The contents of Al_2_O_3_, Fe_2_O_3_ and Rb (Table 3, Fig 4) in paleosol layers (L_1-S1_ and L_1-S2_) were significantly higher than the contents of Al_2_O_3_, Fe_2_O_3_ and Rb in loess layers (L_1-1_, L_1-2_, L_1-3_), but lower than these of S_0_ (Table 2), and the contents of Na_2_O, CaO, and Sr exhibit the opposite trend, which indicates that the weak paleosol had undergone obvious changes in pedogenesis. However, its weathering intensity is weaker than that of S_0_.

Based on the analysis of magnetic susceptibility, grain size, and geochemical elements, it can be concluded that the chemical weathering degree of S_0_ is obviously higher than that of L_1_ (L_1-1_, L_1-2_, L_1-3_), and the chemical weathering degree of L_1-S1_ and L_1-S2_ is also higher than that of L_1_ (L_1-1_, L_1-2_, L_1-3_).

### 5.2. MIS 3 and LGM climate fluctuations in the TJW profile

The above analysis shows that the climate in Yunxian county can be divided into four stages, with cold dry (55–11.5 ka) → from cold and dry to warm and humid (11.5–8.5 ka) → warm and humid (8.5–3.1 ka) → from warm and humid to cold and dry (3.1–1.5 ka).

**Fig 5:**
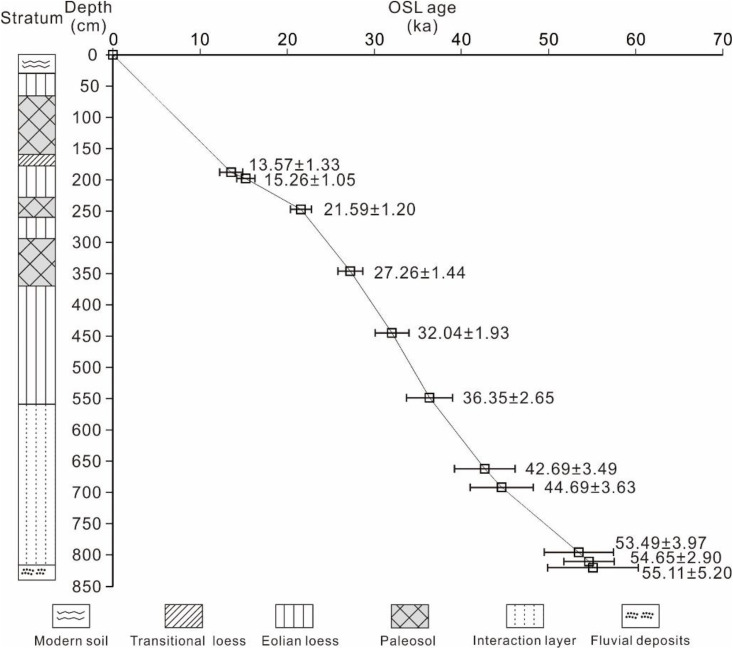
Pedostratigraphic subdivisions, OSL age/depth curve in the loess-paleosol sequences at the TJW profile in the upper Hanjiang River valley [17].

**Fig 6:**
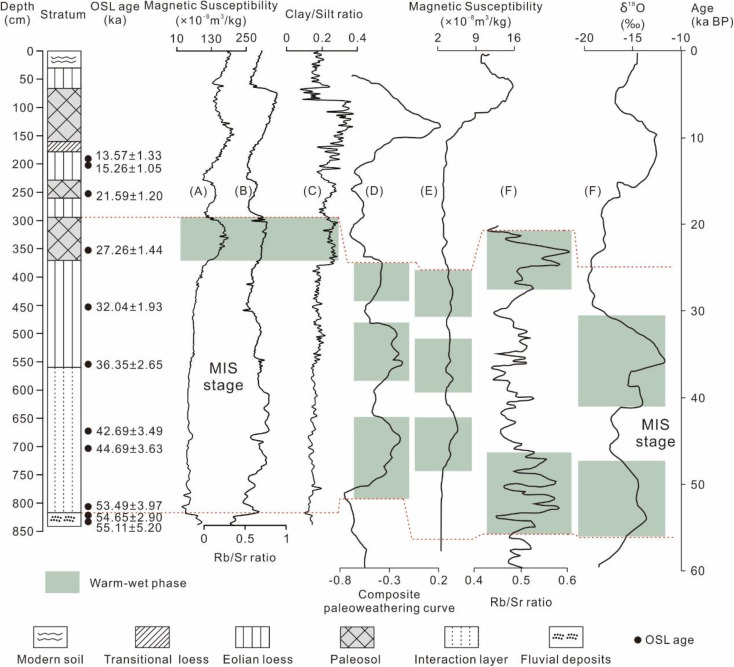
(A. B, C) The variations of the magnetic susceptibility, Rb/Sr ratio, clay/silt ratio in the TJW profile in the upper Hangjiang River valley. **(D)** The stacked paleo-weathering curve of the loess profiles at Weinan, Luochuan, and Yichuan sites [3]. **(E)** The magnetic susceptibility variation of the Yuanbao profile in Linxia City [7]. **(F)** The Rb/Sr ratio variation of the Huining profile in Gansu Province [8]. **(G)**. The content of δ^18^O in Guliya ice core [47].

The climate during the L_1_ stage (55–11.5 ka) was dry and cold, dominated by strong winter winds. It is worth noting that two layers of L_1-S2_ and L_1-S1_ in L_1_ layer indicate two warm and humid periods, which occurred approximately at 27.26 ± 1.44 ka and 21.59 ± 1.20 ka respectively (Fig 5, 6). Considering the location and dating error of the samples, it can be inferred that the formation time of the two layers is about 28.5–21.5 ka BP, indicating that the two relatively warm and humid events occurred in the Hanjiang River region during this period, and there were obvious fluctuations of cold and warm. The formation time of L_1-S2_ (27.26 ± 1.44 ka) was about the same as that of late MIS 3 in the world, indicating that the warm and humid climate of late MIS 3 was also present in the Hanjiang River region. The formation of L_1-S1_ (21.59 ± 1.20 ka) may indicate climatic fluctuations during the last glacial maximum. Based on multi-index analysis, the degree of warmth and humidity during these two periods was slightly lower than that during the S_0_ formation stage (Fig 3, Fig 4). The climate during the L_t_ stage (11.5–8.5 ka) changed from dry and cold to warm and humid. The S_0_ stage (8.5–3.1 ka) was the warmest and most humid conditions, as indicated by the intensity of weathering recorded in the TJW profile. The climate in the L_0_ stage (after 3.1 ka) changed from warm and humid to dry and cold.

### 5.3. MIS 3 climate events and regional comparison recorded in TJW profile

Climate change trends towards relatively warm and humid conditions during MIS 3 have been recorded in many other regions of the world [44,45]. For instance, in the northwest region where there are significant differences in precipitation, temperature and the upper reaches of the Hanjiang River, similar climate fluctuation phenomena similar to those observed in the MIS3 stage of the TJW profile were also recorded [7,8]. According to paleo-weathering curves of loess profiles in Luochuan and other places, MIS 3 can be roughly divided into three warm and wet stages and two cold and dry stages, but the temperature and humidity degree of the late stage is weaker than that of the early stage [3] (Fig 6). The study of Yuanbao profile in Linxia county (Fig 6) shows that climate in this period can be divided into five stages, with high temperature and humidity (56.1–42.2 ka) → cold dried (42.2–39.3 ka) → mild temperature (39.3–33.1 ka) → cold dry (33.1–31.0 ka) → medium isothermal wet (31.0–25.0 ka), the temperature and humidity of the late stage in MIS 3 was lower than that of the early stage [7]. The MIS 3 climate recorded from the Huining profile in Gansu Province (Fig 6) can be divided into three stages: low temperature and humidity (55.5–45.9 ka) → dry cold (45.9–27.3 ka) → high temperature (27.3–23.8 ka), the temperature and humidity degree of the late period is higher than that of the early period [8]. Although there are climatic differences between the northwest region and the upper reaches of the Hanjiang River, due to the influence of the regional atmospheric circulation pattern, the overall soil formation process remains similar. In addition, ice-cores also recorded the climate fluctuation phenomena during the MIS 3 period [46–48]. For example, δ^18^O studies from Greenland ice-cores indicate that climate during the MIS 3 is anomalous and warm [46,47]. The Gurilya ice core also records that there were two periods of climate warming during MIS 3 [48]. Zheng et al selected three sedimentary cores with relatively high and continuous sedimentation rates in the north, west and south of the South China Sea as their research subjects. They revealed that there were multiple rapid climate change events during the MIS 3 period [49]. Johan et al conducted a study on the sediments of the Scandinavian Peninsula, and the results showed that from 55 ka to 35 ka, it was an ice retreat period, and then the ice sheet began to expand [50]. It indicates that there were fluctuations in temperature during the MIS 3 period.

In conclusion, the MIS 3 recorded in the TJW profile represents a specific response of this region to global climate change. It is found that climate change records of MIS 3 period are not identical among different sites. It is speculated that these differences mainly exist in the following aspects: First, the upper reaches of the Hanjiang River are located in the south of the Qinling Mountains, which is the intersection area of various monsoon/air masses (East Asian summer monsoon, Indian monsoon), and its hydrothermal configuration is different from that of the Loess Plateau dominated by the pure East Asian monsoon. The second is the terrain effect: the barrier effect of Qinling Mountains may weaken the influence of cold air in the north, and may also modulate the water vapor transport. The third is the sensitivity of alternative indicators: discuss the possible differences in the climatic significance of the same indicator (such as magnetic susceptibility) in different climatic regions. The relevance of our findings to global records such as the Guliya ice core suggests that it is both a response to global signals and has regional characteristics.

## 6. Conclusion

An in-depth survey was carried out on the first terrace of the Hanjiang River valley, and a loess-paleosol profile was identified. Multi-proxy analysis was used to reconstruct the climate changes recorded in this profile during MIS 3. The main conclusions are as follows:

1. Chemical weathering of S_0_ is the strongest in the formation period, and the weathering of Malan loess is weak in the formation period, but the weathering of Malan loess is stronger in the depths of 228–260 cm and 294–370 cm, and the pedogenesis is obvious. It belongs to weak a paleosol layer (L_1-S1_ and L_1-S2_).
2. Malan Loess layer indicates that the climate was dry and cold. Weak paleosol layers (L_1-S1_ and L_1-S2_) suggest that the climate was not continuously dry and cold during late MIS 3. There are two periods of warm and humid climate during about 28.5–21.5 ka. Loess (L_t_) indicates that the climate changes from dry-cold to warm-wet.
3. There are significant differences in the manifestation and intensity of the relative warm and humid climate in the MIS 3 between the north and south of China. The upper reaches of the Hanjiang River may record the unique information of the interaction between the East Asian monsoon system and the middle and low latitude climate system.

## Supporting information

S1 File Dataset of Magnetic Susceptibility and Grain Size from the Tuojiawan Profile. (XLSX)

S2 File Dataset of Geochemical Elements from the Tuojiawan Profile. (XLSX)
